# An ortholog of the *Leptospira interrogans* lipoprotein LipL32 aids in the colonization of *Pseudoalteromonas tunicata* to host surfaces

**DOI:** 10.3389/fmicb.2014.00323

**Published:** 2014-07-03

**Authors:** Melissa Gardiner, David E. Hoke, Suhelen Egan

**Affiliations:** ^1^Centre for Marine Bio-Innovation, School of Biotechnology and Biomolecular Sciences, The University of New South WalesSydney, NSW, Australia; ^2^Department of Biochemistry and Molecular Biology, Monash UniversityClayton, VIC, Australia

**Keywords:** LipL32, seaweed/s, marine bacteria, *Pseudoalteromonas*, host-microbe interaction, bacterial attachment, algae

## Abstract

The bacterium *Pseudoalteromonas tunicata* is a common surface colonizer of marine eukaryotes, including the macroalga *Ulva australis.*Genomic analysis of *P. tunicata* identified genes potentially involved in surface colonization, including genes with homology to bacterial virulence factors that mediate attachment. Of particular interest is the presence of a gene, designated *ptlL32*, encoding an ortholog to the *Leptospira* lipoprotein LipL32, which has been shown to facilitate the interaction of *Leptospira* sp. with host extracellular matrix (ECM) structures and is thought to be an important virulence trait for pathogenic *Leptospira*. To investigate the role of PtlL32 in the colonization by *P. tunicata* we constructed and characterized a Δ*ptlL32* mutant strain. Whilst *P. tunicata* Δ*ptlL32* bound to an abiotic surface with the same capacity as the wild type strain, it had a marked effect on the ability of *P. tunicata* to bind to ECM, suggesting a specific role in attachment to biological surfaces. Loss of PtlL32 also significantly reduced the capacity for *P. tunciata* to colonize the host algal surface demonstrating a clear role for this protein as a host-colonization factor. PtlL32 appears to have a patchy distribution across specific groups of environmental bacteria and phylogenetic analysis of PtlL32 orthologous proteins from non-*Leptospira* species suggests it may have been acquired via horizontal gene transfer between distantly related lineages. This study provides the first evidence for an attachment function for a LipL32-like protein outside the *Leptospira* and thereby contributes to the understanding of host colonization in ecologically distinct bacterial species.

## Introduction

Macroalgae, or seaweeds, are important ecosystem engineers in temperate marine environments and are a rich source of biologically active compounds (Egan et al., 2008, 2013b). The surface-associated microbial community (SAMC) that rapidly colonize the alga have important roles for normal morphological development, nutrient supply, and defense against unwanted colonizers (Goecke et al., 2010; Wahl et al., 2012; Hollants et al., 2013). Furthermore, the SAMC can influence the health of the host alga and members of this community have the potential to function as opportunistic pathogens (Gachon et al., 2010; Egan et al., 2013a). The composition of the SAMC for many macroalgal species has been well studied and includes both generalist epibionts and host-specific taxa that are distinct from the surrounding seawater (Longford et al., 2007; Tujula et al., 2010; Burke et al., 2011; Lachnit et al., 2011). In contrast, there remains a paucity of knowledge regarding the specific mechanisms that facilitate interaction of the SAMC with the host alga (Egan et al., 2013a).

Attachment is considered a key stage in host colonization, and facilitates the progression of both commensal and pathogenic bacterial-host interactions (Kline et al., 2009; Petrova and Sauer, 2012; Bogino et al., 2013). The abundance of specialized adhesins and pili encoded in the genomes of seaweed-associated bacteria indicates this is similarly an important aspect of macroalgal epibiosis (Thomas et al., 2008; Fernandes et al., 2011; Thole et al., 2012). The marine bacterium *Pseudoalteromonas tunicata* was originally isolated from the tunicate *Ciona intestinalis* and has since been well studied as a surface colonizer of the alga *Ulva australis* (Holmström et al., 1998; Rao et al., 2005, 2006). Attachment of *P. tunicata* to biotic and abiotic surfaces occurs within 2 h of contact, and cells proceed into biofilm formation within 24 h through the production of differentiated mushroom-shaped microcolonies (Mai-Prochnow et al., 2004; Dalisay et al., 2006). With the exception of a MSHA-like pili that has been demonstrated to play a role in the attachment of the bacterium to both abiotic and host surfaces (Dalisay et al., 2006), there is a lack of experimental data on the specific factors that mediate host colonization in this bacterium. Genome analysis of *P. tunicata* has identified genes with homology to putative colonization factors, lipoproteins, pili and outer membrane proteins (OMP) (Thomas et al., 2008). A number of these genes have homology to factors that mediate specific interactions with host cells in other bacteria. One example, designated *ptlL32* (locus tag PTD2_05920) encodes for a protein with 47% identity to the *Leptospira* MSCRAMM (microbial surface components recognizing adhesive matrix molecules) lipoprotein, LipL32.

*Leptospira* species are the causative agent of the endemic zoonotic infection, leptospirosis (reviewed in Levett, 2001; Adler and de la Pena Moctezuma, 2010). LipL32 is highly conserved in pathogenic *Leptospira* species (with an average 98% amino acid identity) where it is the most abundantly expressed lipoprotein (Haake et al., 2004; Dey et al., 2007; Adler et al., 2011). The absence of LipL32 in saprophytic *Leptospira* strains and its ability to bind to extracellular matrix (ECM) structures suggest that LipL32 plays a major role in host-cell attachment during mammalian infections (Hauk et al., 2008; Hoke et al., 2008; reviewed in Murray, 2013). However the precise involvement of LipL32 in *Leptospira* pathogenesis remains unclear as recent studies using a *L. interrogans lipL32* transposon mutant failed to demonstrate a direct role for this protein in infection models (Murray et al., 2009).

*P. tunicata* rapidly attaches to ECM structures (Hoke et al., 2011) and the tunicate, *C. intestinalis*, a natural host of *P. tunicata*, possesses the genes necessary for ECM synthesis, including those encoding for collagen type IV, fibronectin, laminin, and nidogen (Huxley-Jones et al., 2007). In addition, *Ulva liza*, a close relative of *U. australis*, possesses the genes encoding ECM-like proteins, including collagen (Stanley et al., 2005). Interestingly, recombinantly produced PtlL32 bind ECM structures in a manner analogous to LipL32 from *Leptospira* sp. (Hoke et al., 2008). Moreover heterologously expressed PtlL32 is immunologically cross reactive with *Leptospira* LipL32 antibodies (Hoke et al., 2008) and the amino acid sequence across the characteristic calcium-binding and putative polypeptide binding regions of LipL32 is conserved in the two proteins (Hauk et al., 2009). This biochemical information suggests that PtlL32 is an ECM-binding protein, however the biological function and importance for *P. tunicata* is not established. Moreover prior to the identification of this LipL32 ortholog in *P. tunciata*, it was widely believe that LipL32 proteins were unique to pathogenic *Leptospira* spp. (Murray, 2013), raising the question as to the origin of PtlL32 and its' prevalence in other environmental bacteria. Here we use a combination of allelic exchange mutagenesis, attachment assays, colonization experiments, and phylogenetic analysis to demonstrate a role for this conserved lipoprotein in facilitating bacterial interaction with host surfaces and provide evidence that suggests LipL32-proteins may have been acquired via horizontal gene transfer from an environmental origin. This study provides the first experimental evidence for a function of LipL32-like proteins outside of the *Leptospira* genus and builds upon our current understanding of the traits that drive host colonization in marine bacteria.

## Materials and methods

### Allelic exchange and complementation of *p. tunicata ptlL32*

A *P. tunicata ptlL32* allelic replacement mutant strain was generated using the Gene Splicing by Overlap Extension (SOE) PCR strategy (Horton, 1995) coupled with bi-parental conjugation and homologous recombination as described previously for the mutagenesis of *P. tunicata* (Egan et al., 2002; Mai-Prochnow et al., 2004). Briefly, the first section of the *ptlL32* gene was amplified from wild type (WT) genomic DNA with primers, first-forward, 5′ ATG AAA ATC AAA CTG GTC GTG G 3′; and first-reverse, 5′ CTG GTT TCG CTA AAT CAC CCA C 3′. In a separate PCR reaction, the second section was amplified with primers, second-forward, 5′ CAG ACA AAT TAA AAG CCG ATA AAG; and second-reverse, 5′ TTA TTT ATT GAC TGC TTT ATG TAA C 3′. A kanamycin resistance (kan^*R*^) cassette was amplified from the plasmid pACYC177 (Table 1) using the following primers: kan^*R*^ forward, 5′ GAT TTA TTC AAC AAA GCC ACG 3′; kan^*R*^ reverse, 5′ ATT TAT TCA ACA AAG CCG CC 3′. A recombinant *ptlL32* knockout fragment (*ptlL32*::kan^*R*^) was constructed using SOE-PCR (Horton, 1995) by “PCR splicing” the overhangs on the first and second PCR products onto the 5′ and 3′ ends of the kan^*R*^ cassette, respectively. Following PCR amplification of *ptlL32*::kan^*R*^ using the first-forward and second-reverse primers, the fragment was introduced into the *EcoRI* site of the suicide vector pGP704 (Table 1) (Miller and Mekalanos, 1988) to generated the vector pLP704. Standard electroporation techniques (Ausubel et al., 1994) were used to transfer pLP704 into *E. coli* SM10. The *P. tunicata* Δ*ptlL32* strain was constructed using allelic exchange by conjugation of the recombinant *E. coli* SM10 pLP704 strain with *P. tunicata* WT (str^*R*^) (Table 1) (according to the method described in Egan et al., 2002). Exconjugants with the *ptlL32*::kan^*R*^ fragment inserted into the chromosome by homologous recombination were selected using VNSS (Marden et al., 1985) agar plates supplemented with streptomycin (200 μg ml^−1^) and kanamycin (85 μg ml^−1^). PCR confirmation of the recombination event was performed with a forward primer that target a region upstream of *ptlL32* in the WT chromosome, 5′ AAG CAT CCA GTG TGC AGT CG 3′, and the kan^*R*^ reverse primer.

**Table 1:** **Plasmids and bacterial strains used in this study**.

**Strain or plasmid**	**Relevant genotype^#^**	**References**
***E. coli***		
SM10 λ pir	RP4-2-Tc::Mu, π replicase (pir); str^*s*^ kan^*s*^	Simon et al., 1983
SM10 pLP704	pGP704:: *ptlL32::kan^*R*^ amp*	This study
SM10 pBBR1L	pBBR1 MCS5::*ptlL32 gen*	This study
SM10 pCJS10	pCJS10 *cat*	Dalisay et al., 2006
***P. tunicata* D2**		
WT str^R^	Spontaneously resistant to streptomycin	Egan et al., 2002
GFP-labeled WT str^R^	pCJS10 *cat str^R^*	Dalisay et al., 2006
Δ*ptlL32*	*ptlL32* knockout mutant; *str^R^, ptlL32*::*kan^*R*^ amp*	This study
CΔ*ptlL32*	Complemented *ptlL32* knockout mutant; *str, ptlL32*::*kan^*R*^ amp*; pBBR1 MCS5*ptlL32 gen*	This study
GFP-labeled Δ*ptlL32*	*str^R^, ptlL32* knockout mutant; *ptlL32*::*kan^*R*^ amp;* pCJS10 *cat*	This study
**PLASMIDS**		
pBBR1 MCS5	Broad host range mobilizable vector, *gen*	Kovach et al., 1995
pBBR1 MCS5::*ptlL32*	Complementation vector containing intact *ptlL32*	This study
pCJS10-GFP	RSF1010 broad host range backbone, *gfp*mut3, *cat*	Rao et al., 2005
pGP704	Suicide vector, R6K ori, mob, *amp*	Miller and Mekalanos, 1988
pLP704	pGP704 with *ptlL32*::*kan^*R*^* knockout fragment	This study
pACYC177	Cloning vector, *amp kan^*R*^*	Chang and Cohen, 1978

# str^S^, streptomycin sensitive; kan^S^, kanamycin sensitive; str^R^, streptomycin resistance; kan^*R*^, kanamycin resistance; tet, tetracyclin resistance; amp, ampicillin resistance; cat chloramphenicol resistance; gen, gentamycin resistance.

To complement the Δ*ptlL32* strain, the *ptlL32* gene and surrounding potential promoter and terminator regions was amplified from WT genomic DNA with primers: WT forward, 5′ GCA ATA GCT TTC TTT GTT CCT C 3′; WT reverse, 5′ GAC ACA TCA GCA TCA CTC AC 3′. The *ptlL32* PCR product was ligated into the *SmaI* site of the broad host range plasmid, pBBR1 MCS5 (Kovach et al., 1995) to generate the complementation plasmid, pBBR1L. Standard electroporation was used to transfer pBBR1L into an *E. coli* SM10 donor strain (Ausubel et al., 1994), before bi-parental conjugation of the donor strain with *P. tunicata* Δ*ptlL32* as described previously (Egan et al., 2002). *P. tunicata* Δ*ptlL32* ex-conjugants with *ptlL32* complemented *in trans* were selected for on VNSS agar plates supplemented with gentamycin (50 μg ml^−1^), kanamycin and streptomycin, and verified by PCR using the WT forward primer, and the gen resistance cassette reverse primer 5′ GCGGCGTTGTGACAATTT 3′. The confirmed complemented Δ*ptlL32* strain was named CΔ*ptlL32*.

### Bacterial attachment to polystyrene and matrigel™ basement membrane matrix

The attachment of *P. tunicata* Δ*ptlL32* to polystyrene was compared to the WT using a cell adhesion assay. The bacterial strains were grown at 28°C for 24 h shaking in VNSS supplemented with the appropriate antibiotics (Table 1). The cell suspension was centrifuged (6000 × g, 5 min), washed twice and resuspended in 1 ml of sterile NSS (Marden et al., 1985) at Abs_600 nm_ = 1 (~10^9^ CFU ml^−1^). Fifty microliters of cell suspension for each strain was added to triplicate wells of a polystyrene Costar® 96 well plate (Corning™). The plate was incubated for 6 h at 28°C with gentle shaking before non-adherent cells were removed by rinsing the wells six times with sterile PBS. Twenty-five microliters of a 200 μg ml^−1^ solution of trypsin were added to each well and the plate incubated at 37°C for 5 min to allow for cell detachment. Detached cells were then counted by dark field microscopy in a Helber™ bacterial counting chamber (Hawksley, Sussex, UK). Attached bacteria from a total 80 small squares (chosen based on the results of a random number generator) on the counting chamber were enumerated for each of the replicates. The experiment was performed in triplicate for each strain on three independent days. Statistical analysis was performed using SYSTAT 13 (SYSTAT Software Inc., USA) and significance was assessed using an unpaired Students two-tailed *t*-test.

To assess the attachment of Δ*ptlL32* to ECM, the assay outlined above was modified according to the protocol described in Hoke et al. (2008). The ECM preparation used in this study, BD Matrigel™ Basement Membrane Matrix (BD Biosciences, USA), is derived from Engelbreth-Holm-Swarm (EHS) mouse sarcoma cells, and is composed mainly of laminin, entactin, collagen, and mammalian growth factors (Vukicevic et al., 1992; Hughes et al., 2010). The wells of a 96 well plate were coated with 50 μL of BD Matrigel™ and incubated at 4°C overnight. The wells were then washed three times with PBS to remove excess ECM. The *P. tunicata* bacterial strains (Table 1) were grown, washed, and resuspended as described for the polystyrene adhesion experiment above. Fifty microliters of cell suspension for each strain was added to triplicate wells of a plate coated with BD Matrigel™, and incubated for 2 and 6 h at 28°C with gentle shaking. Non-adherent cells were then removed and detached cells enumerated as described for the polystyrene adhesion experiment. Significance was assessed using a One Way analysis of variance (ANOVA).

### Bacterial attachment to *u. australis*

To assess the adhesion of *P. tunicata* Δ*ptlL32* to a living host surface an attachment assay to *U. australis* was performed as described previously (Dalisay et al., 2006). The *U. australis* samples were collected from Clovelly Bay, Sydney Australia and processed immediately. The algal samples were rinsed four times with 50 ml of autoclaved seawater and thallus sections of approximately 6 mm were excised from the mid thalli using a sterile scalpel. The algal surface was then cleaned to remove the majority of epiphytic bacteria. Briefly individual samples were swabbed with sterile cotton tips, containing 0.012% NaOCl for 5 min, and incubated for 24 h in an antibiotic mixture that consisted of ampicillin (300 μg ml^−1^), polymyxin (30 μg ml^−1^), and gentamycin (60 μg ml^−1^). The alga thalli were then incubated for 1 h in 50 ml of 0.2 μm filtered-seawater to remove residue chemicals.

*P. tunicata* Δ*ptlL32* was labeled with green florescent protein (GFP) by introducing the broad host range plasmid pCJS10 as described previously for the WT strain (Dalisay et al., 2006) using bi-parental conjugation with an *E. coli* SM10 donor strain (Egan et al., 2002) (Table 1). GFP-labeled Δ*ptlL32* ex-conjugants were grown on VNSS agar plates supplemented with chloramphenicol (18 μg ml^−1^) and confirmed by visual inspection under the GFP filter cube of an epifluorescence microscope (DM-LB Leica). For the *U. australis* attachment assays, GFP tagged WT (Dalisay et al., 2006) and Δ*ptlL32* (Table 1) were grown in VNSS supplemented with chloramphenicol for 24 h at 28°C with shaking. One milliliter of culture was harvested by centrifugation (6000 × g, 2 min), washed three times with NSS, and resuspended at an Abs_600 nm_ of 0.3. A single thallus section was placed in a well of a 6 well plate (Corning™) containing 1 ml PBS and 1 ml of bacterial culture, and incubated for 6 h with gentle shaking.

After 6 h incubation the algal samples were rinsed three times in 2 ml PBS to remove loosely attached cells and visualized immediately using a Confocal Laser Scanning Microscope (Olympus Fluoview FV1000) under 40× oil magnification and 488 nm excitation. Seven images were taken randomly across each sample and the images were analyzed using ImageJ (Schneider et al., 2012). The number of GFP-fluorescing cells attached to the surface of the algae per mm^2^ was quantified using the “analyze particles” function in ImageJ and the results were plotted using GraphPad Prism 6. In addition the number of microcolonies was manually counted (where >10 cells clustered together was classified as a microcolony) and the results plotted using GraphPad Prism 6. The assay was replicated four times in triplicate for each bacterial strain and data analyzed as described for the polystyrene adhesion experiment.

### Sequence retrieval and protein alignment of ptlL32 orthologs

Bacterial genomes were searched for orthologs to the *P. tunicata* gene PtlL32 using a blastp search tool of both the UniProt database (http://www.uniprot.org/) and the non-redundant database of the National Centre for Biotechnology Information (NCBI) in April 2014 (Altschul et al., 1990). All non-*Leptospira* orthologs with greater than 40% amino acid sequence identity to PtlL32 from *P. tunicata* (Table 2) and a further five sequences from representative *Leptospira* species were selected (*L. interrogans*, ADJ95774; *L. kirschneri*, AAF60198; *L. broomii*, EQA47312; *L. borgpetersenii*, ABJ79303*; L. santarosai*, AAS21795). All sequences were aligned with ClustalX using the default parameters (Larkin et al., 2007) and the resulting alignment curated with Gblocks to remove gap positions (Talavera and Castresana, 2007). The resulting alignment of 102 amino acid positions was then subject to maximum-likelihood analysis using PhyML 3.0 with the default LG substitution model and 100 bootstraps (Criscuolo, 2011). Trees were visualized using Dendroscope (Huson and Scornavacca, 2012). Information on the phylogeny and isolation of bacterial strains was obtained from published data and using the genome browser in the Integrated Microbial Genome (IMG) browser (https://img.jgi.doe.gov/cgi-bin/er/main.cgi) in April 2014 (Markowitz et al., 2009).

**Table 2:** **Characteristics of non-*Leptospira* species that possess orthologs of LipL32**.

**NCBI accession**	**Bacterial strain**	**Taxonomic affiliation**	**Isolation source**
EAR27184	*Pseudoalteromonas tunicata*	C: Gammaproteobacteria	Surface of the macroalga *Ulva* spp. (Australia) and the invertebrate *Ciona intestinalis* (Sweden)
		O: Alteromondales	
		F: Pseudoalteromonadaceae	
ERG54672	*Pseudoalteromonas spongiae*	As above	Surface of the sponge *Mycale adhaerens* in Hong Kong
ERG42655	*Pseudoalteromonas rubra*	As above	Mediterranean seawater of the coast of France
ESP92640	*Pseudoalteromonas luteoviolacea*	As above	Surface of the coral *Montastrea annularis* in reef water off the coast of Florida, USA
ADZ89577	*Marinomonas mediterranea*	C: Gammaproteobacteria	Mediterranean seawater from the southeastern coast of Spain
		O: Oceanospirillaceae	
		F: Oceanospirillales	
EDM65737	*Moritella* sp. PE36	C: Gammaproteobacteria	Deep ocean waters of the Pacific Ocean San Diego, USA
		O: Alteromondales	
		F: Moritellaceae	
ADV49486	*Cellulophaga algicola*	P: Bacteroidetes	Surface of a sea ice-chain forming pennate diatom, *Melosira* sp. in the Eastern Antarctic coastal zone
		C: Flavobacteria	
		O: Flavobacteriales	
		F: Flavobacteriaceae	
ADY29818	*Cellulophaga lytica*	As above	Beach mud in Limon, Costa Rica
CDF79332	*Formosa agariphila*	As above	Surface of the green alga *Acrosiphonia sonder* isolated from the Sea of Japan
EDM44616	*Ulvibacter* sp. SCB49	P: Bacteridetes	Surface waters of the Pacific Ocean Southern California Bight, USA
		C, O, F: Unclassified	
AEE17958	*Treponema brennaborense*	P: Spirochaetes	Ulceritive skin lesion on a bovine foot infected with digital dermatitis, Germany
		C: Spirochaetia	
		O: Spirochaetales	
		F: Spirochaetaceae	

## Results and discussion

### PtlL32 is not required for attachment to abiotic surfaces, however contributes to the attachment of *p. tunicata* to host surfaces

**Figure 1:**
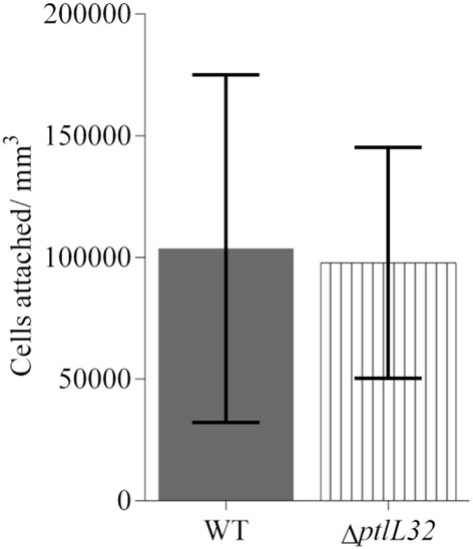
**Attachment of *P. tunicata* WT (solid fill) and ΔptlL32 (vertical stripes) to a polystyrene well plate after 6 h of incubation**. The numbers of cells attached per mm^3^ were determined using direct counts of detached bacteria in a Helber bacterial counting chamber. Significance was assessed using the Students unpaired, two-tailed *t*-test (*p* > 0.8). Error bars represent standard deviation.

To investigate the role of *ptlL32* in attachment to abiotic surfaces a *ptlL32* knock-out mutant (Δ*ptlL32)* was constructed and was compared to the WT *P. tunicata* strains for its ability to adhere to a polystyrene surface. After 6 h incubation there was no significant difference (*p* > 0.8) between the number of cells attached for the Δ*ptlL32* strain compared to WT (Figure 1). These data demonstrate that a mutation in *ptlL32* has no immediate impact on the ability of *P. tunicata* to attach to abiotic surfaces.

**Figure 2:**
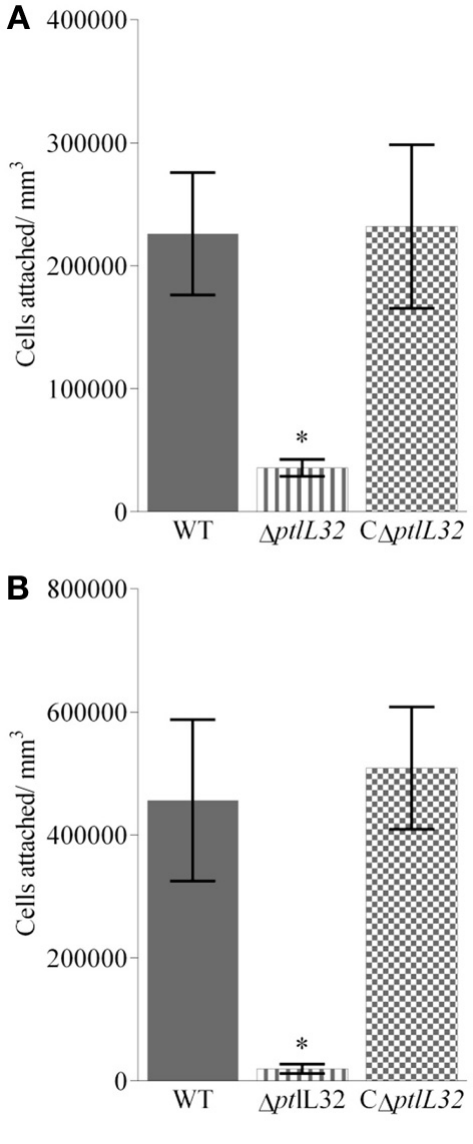
**Attachment to Matrigel™ after 2 h (A) and 6 h (B) for ΔptlL32 (horizontal stripes) compared to WT (solid fill) and C ΔptlL32 (small squares)**. The number of cells attached per mm^3^ for each strain was estimated using direct counts of detached bacteria in a Helber bacterial counting chamber. Averages are shown as columns with *n* = 9. Error bars represent standard deviation. ^*^Indicates significance with a *p* < 0.001 using an ANOVA.

Attachment of the *P. tunicata* Δ*ptlL32* strain to ECM structures was also compared to WT and CΔ*ptlL32*, at times that have been characterized as early (2 h) and late (6 h) stages of irreversible attachment in other bacterial species (Hinsa et al., 2003; Palmer et al., 2007; Li et al., 2012). Figures 2A,B show the average numbers of cells attached per mm^3^ for the three strains after 2 and 6 h, respectively. The mutant strain Δ*ptlL32* exhibited 10-fold reduction (*p* < 0.001) in attachment compared to WT after both 2 and 6 h. There was also a clear increase in attached cells over time for the WT, but not for the mutant strain (Δ*ptlL32)* (Figure 2). Complementation of *ptlL32 in trans* (strain CΔ*ptlL32*) restored the WT phenotype, excluding polar effects of the knock-out mutant. Together with the observations that mutations in *ptlL32* had no effect on attachment of *P. tunicata* to abiotic surfaces (Figure 1), these data show that Ptlp32 contributes specifically to the ability of *P. tunicata* to adhere to complex biological surfaces.

**Figure 3:**
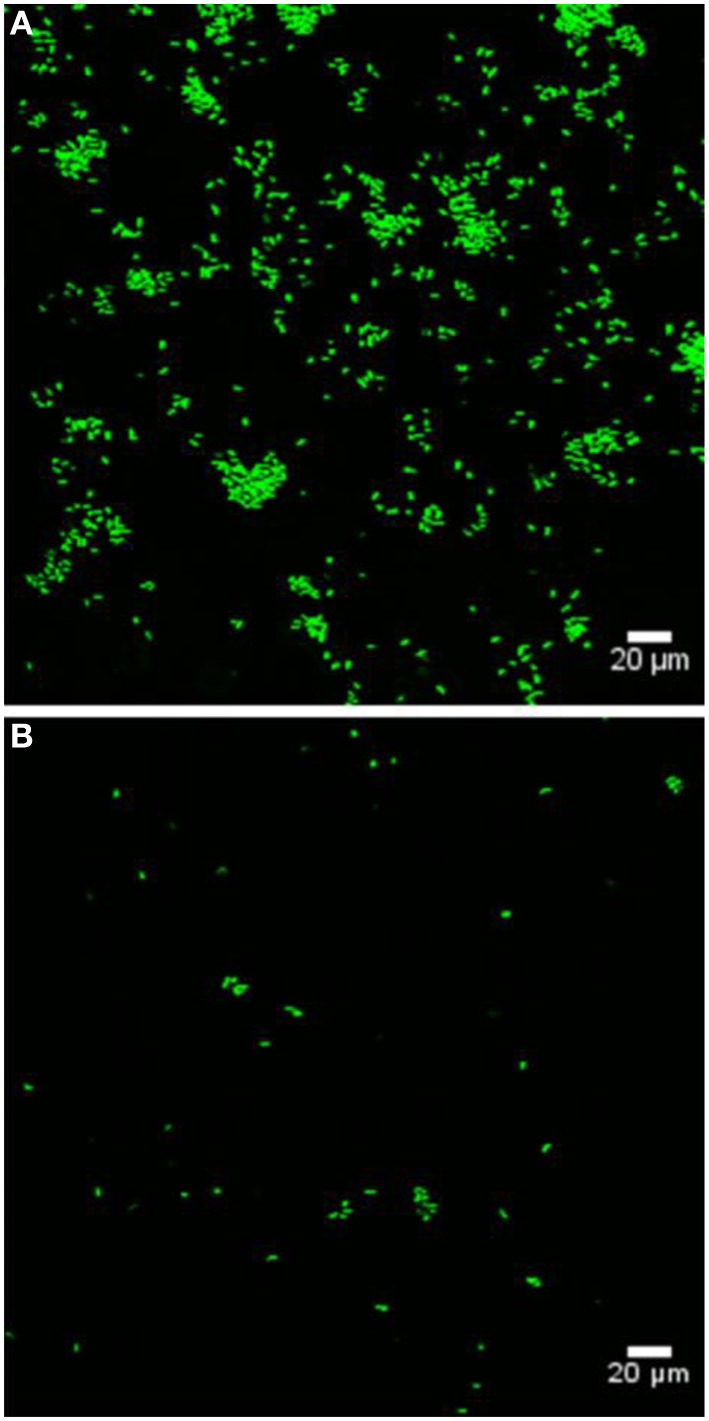
**Representative confocal laser scanning microscopy images of GFP-labeled WT (A) and GFP-labeled ΔptlL32 (B) attached to the surface of *U. australis***. Green fluorescent cells were enumerated using ImageJ software. Images were captured using a FV1000 confocal laser-scanning microscope. Scale bar represents 20 μm.

**Figure 4:**
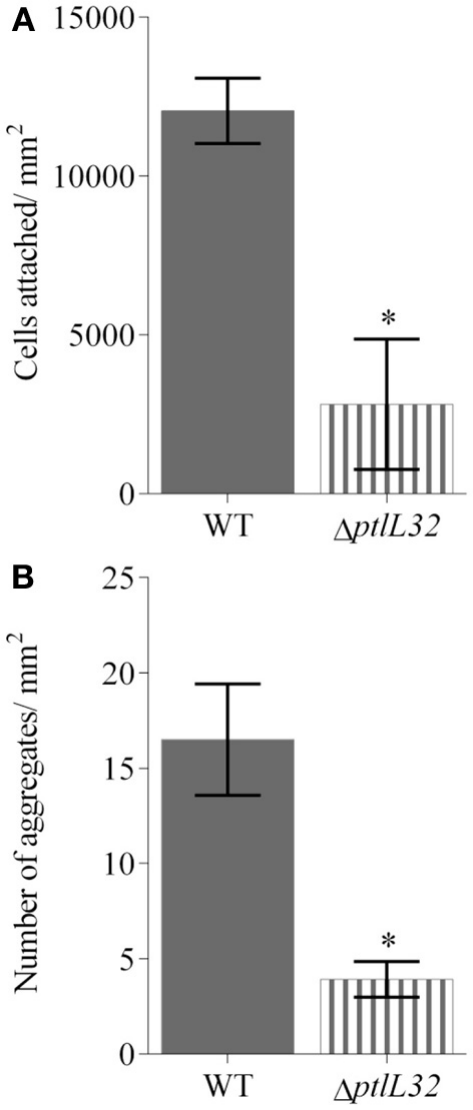
**Average number of cells attached to (A), and cell aggregates (defined as a tight cluster of >10 cells) (B) on *U. australis* for GFP-labeled WT (solid fill) and GFP-labeled ΔptlL32 (horizontal stripes) after 6 h incubation**. The number of green fluorescing cells or aggregates in each field of view was counted using ImageJ and an average expressed for cells per mm^2^. Error bars represent standard deviation. Error bars represent standard deviation at 45 replicates and ^*^ denotes a significant difference with a *p* < 0.001 using the unpaired Students two-tailed *t*-test.

Bacterial colonization of various marine eukaryotes has been demonstrated in other non-algal systems to be mediated by surface-specific adhesins (Mueller et al., 2007; Bulgheresi et al., 2011; Stauder et al., 2012); and we hypothesize that PtlL32 may facilitate adhesion to ECM-like surfaces in the marine environment, including, but not limited to, its natural host *U. australis.* To further explore this role we assessed the ability of Δ*ptlL32* and the WT *P. tunicata* strains to attach to the surface of *U. australis*. *P. tunicata* Δ*ptlL32* demonstrated a significant reduction in the number of cells attached to the surface of the alga (Figures 3, 4A) compared to WT cells (*p* < 0.001). A reduction in the number of cell aggregates on the surface of *U. australis* in the mutant strain (Figure 4B; *p* < 0.001) also indicates that biofilm maturation may be impaired. The reduced attachment of Δ*ptlL32* cells to the surface of the alga compared to WT suggests that PtlL32 may bind to specific host cell wall components. The major cell wall matrix components for *Ulva* species are cellulose and ulvan (Lahaye and Robic, 2007), however the structural details are unknown (Lahaye and Kaeffer, 1997; Robic et al., 2009). Genetic analysis of *U. linza* identified genes involved in the synthesis of hydroxyproline-rich glycoproteins, including collagen (Stanley et al., 2005), which are homologous to components in the ECM preparation used above (Hughes et al., 2010). *Leptospira* LipL32 has also been shown to bind a number of proteins, including different types of collagen (Hauk et al., 2008; Hoke et al., 2008; Chaemchuen et al., 2011). Therefore, the reduced adhesion of Δ*ptlL32* to both ECM and *U. australis* may be the result of interaction of PtlL32 with host proteins, including different forms of collagen.

The observation that PtlL32 mediates colonization only on biotic surfaces stands in contrast to what has been observed for the MSHA-like pili, which mediates adhesion to both biotic and abiotic surfaces (Dalisay et al., 2006). Changes in the membrane proteome of *P. tunicata* between cells grown on either BSA-coated or ECM-coated surfaces have recently been observed (Hoke et al., 2011) and therefore it is likely that *P. tunicata* utilizes a distinct subset of proteins to adhere to different surfaces. Thus the available data suggests a conserved function for LipL32-like proteins in facilitating interaction with ECM structures (Hauk et al., 2008; Hoke et al., 2008), a novel finding given the absence of an overlapping niche between *P. tunicata* and *Leptospira* species.

### PtlL32 orthologs have a patchy distribution across specific groups of environmental bacteria

Given that PtlL32 is important for mediating host colonization in *P. tunicata* we sought to determine the distribution and relationship of orthologous proteins across other non-*Leptospira* organisms. *In silico* analysis of publically available bacterial genomes revealed sequences similar to PtlL32 in eleven other species, of which five were isolated from eukaryotic hosts, ten are marine bacteria and only the spirochete *Treponema brennaborense* has been associated with disease (Table 2). This distribution is interesting considering that until relatively recently LipL32-like proteins were thought to be unique to pathogenic *Leptospira* (Hoke et al., 2008; Murray, 2013). Given the untapped bacterial diversity in environmental ecosystems these data further suggest that the distribution of LipL32 orthologs may be greater still.

Multiple alignment of the protein sequences revealed a total of 29 amino acids positions that are fully conserved across all sequences; with increased conservation of amino acids at positions 85–107 (Supplementary Material). The regions of sequence conservation also include components of the protein that are postulated to mediate binding of host structures in *Leptospira*, including the polypeptide binding function located in the C-terminal (Supplementary Material, amino acids 188–272) (Hauk et al., 2008; Hoke et al., 2008) and an acidic loop at amino acids 164–178 (AKPVQKLDDDDDGDD) (Hauk et al., 2009; Vivian et al., 2009). These regions of amino acid conservation were previously highlighted in the crystal structures of three non-*Leptospira* LipL32 proteins for their potential role in conferring similar binding properties to the orthologous proteins (Hauk et al., 2009; Vivian et al., 2009).

**Figure 5:**
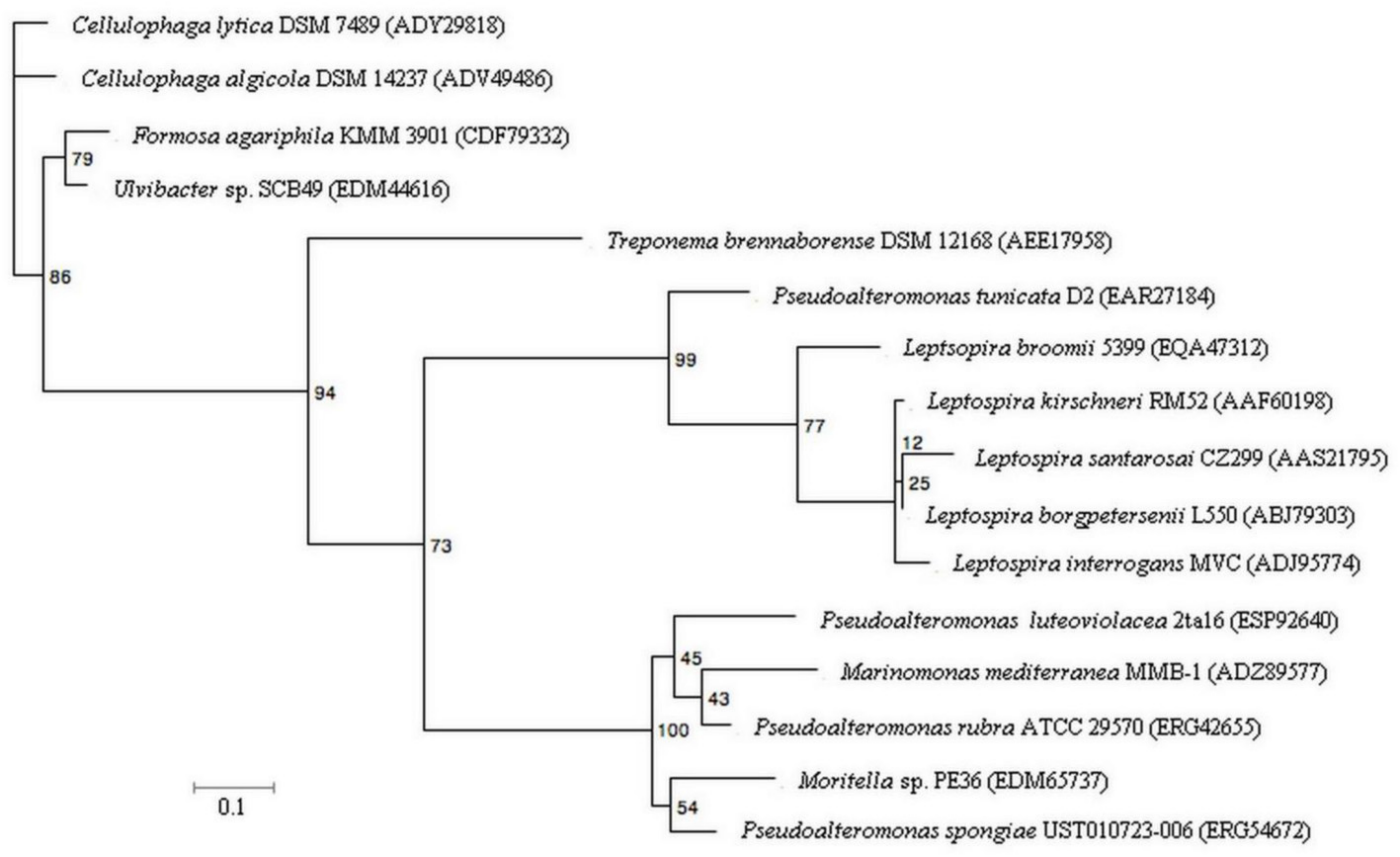
**Maximum-likelihood tree of PtlL32 and orthologous protein sequences in non-*Leptospira* species**. Five representative *Leptospira* LipL32 sequences were included for comparison with the non-*Leptospira* sequence. NCBI GenBank accession numbers for each protein sequence is provided in brackets. Bootstrap values for 100 replicates are shown for each node. The scale bar represents 10% sequence divergence.

Analysis of the phylogenetic relationship between the PtlL32 orthologs provides new insight into its evolutionary origins. Most striking is the observation that the *P. tunicata* sequence clusters with the *Leptospira* and not with those from other *Pseudoalteromonas* species or Gammaproteobacteria (Figure 5). Likewise, a PtlL32 ortholog of *T. brennaborense* (phylum Spirochaetes) does not cluster with the other members of the Spirochaetes (i.e., *Leptospira* spp.) (Figure 5, Table 2). This clustering of LipL32 sequences outside of their taxonomic relatives is in support of an acquisition via horizontal gene transfer (HGT) and/ or maintenance only in the pathogenic *Leptospira* and a selected group of environmental organism. In support of this theory, an examination of the GC content of the *lipL32* genes revealed a deviation from the genomic GC content in examples of *Leptospira lipL32* genes, but not in the non-*Leptospira* orthologs. Given the diversity of the ecosystems in which many of the LipL32 containing strains are found (Table 2) it possible that the *Leptospira* LipL32 has evolved from environmental strains, where it facilitates commensal interactions, to a role in pathogenic interaction with animals. This proposed “dual function” for LipL32 is in line with observations of other environmentally acquired pathogens, where certain colonization traits mediate pathogenesis in a host-associated context, whilst facilitating microbial survival and persistence in the environment (Casadevall et al., 2003; Vezzulli et al., 2008). Indeed an environmental origin of pathogenic *Leptospira* has also been recently suggested and is supported by observations that the closest orthologs to many hypothetical *Leptospira* genes are from environmental rather than pathogenic bacteria (Murray, 2013).

## Conclusion

Understanding of the mechanisms that facilitate bacterial interactions with marine surfaces is poorly understood compared to that of medically relevant systems. Here we have begun to fill this knowledge gap by investigating the mechanisms that facilitate interactions between the marine bacterium *P. tunciata* with its macroalgal host. We found that a *P. tunicata* Δ*ptlL32* strain attached with a greatly reduced capacity to the biotic surfaces (Figures 2–4), a finding that is in line with previous reports indicating that the colonization of *U. australis* by *P. tunicata* involves multiple adhesins (Dalisay et al., 2006; Thomas et al., 2008). Having multiple mechanisms for adhesion may represent a means by which the bacterium can mediate host specificity and/or modify its interaction with the host in response to environmental variability. A degree of functional overlap has also been reported for adhesive structures in a diverse range of bacteria where the apparent redundancy may reflect the ability of these organisms to interact with multiple eukaryotic hosts or tissues (Ramey et al., 2004; Clarke and Foster, 2006; Rodriguez-Navarro et al., 2007; Pruzzo et al., 2008). For example, collagen is also a component of the ECM of the tunicate *C. intestinalis* (Vizzini et al., 2002), since *P. tunicata* has also been isolated from *C. intestinalis* it is possible that PtlL32 also has a function in mediating colonization to both macroalgae and marine invertebrates.

To the best of our knowledge this is the first study to investigate the function for a LipL32 ortholog in an organism outside the *Leptospira* species and the results speak to a conserved function for this protein in mediating association with host cell matrix components. Phylogenetic analysis suggests the *Leptospira* LipL32 was acquired from environmental bacteria and that LipL32 proteins act as “dual function” traits (Casadevall et al., 2003; Casadevall, 2006; Hoke et al., 2008; Murray, 2013) facilitating both pathogenic interactions by *Leptospira* spp. and host colonization by the environmental bacterium *P. tunicata.* This study therefore adds weight to the hypothesis presented by Egan et al. (2013a), that such dual function traits may facilitate interactions between bacteria and macroalgae; a relationship that has important implications for macroalgal health, as well as nutrient cycling, and homeostasis in the temperate ocean environment (Wahl et al., 2012; Egan et al., 2013b).

### Conflict of interest statement

The authors declare that the research was conducted in the absence of any commercial or financial relationships that could be construed as a potential conflict of interest.
