# Supplementary material for: An ortholog of the Leptospira interrogans lipoprotein LipL32 aids in the colonization of Pseudoalteromonas tunicata to host surfaces
Source: Front Microbiol. 2014 Jul 3;5:323. doi: 10.3389/fmicb.2014.00323 (PMC4080168; doi:10.3389/fmicb.2014.00323)
Supplement: Supplementary file 1 [file DataSheet1.DOCX]

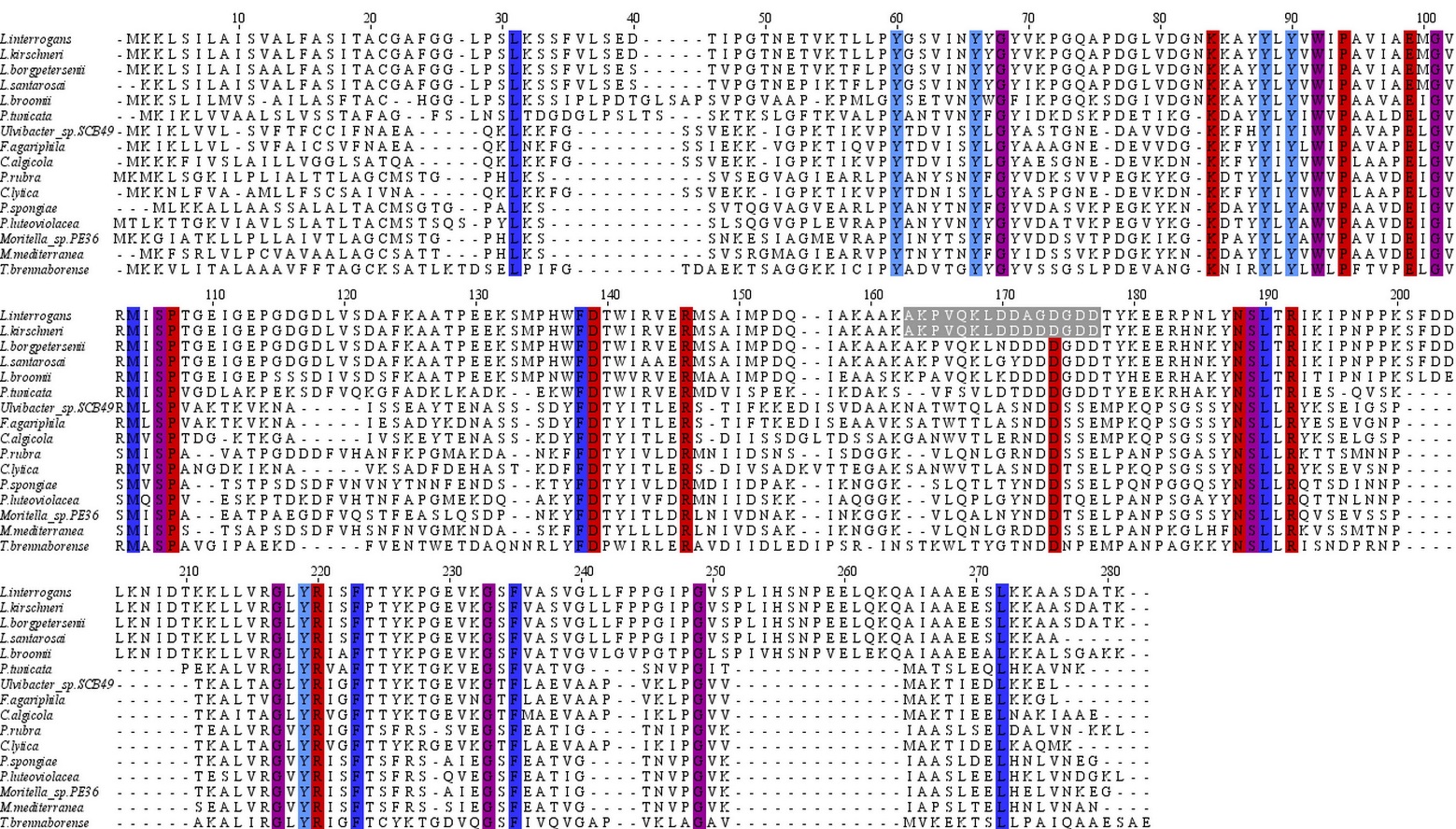


**Supplementary Figure:** ClustalX multiple sequence alignment of the *lipL32* orthologs from the non-*Leptospira* species (Table 2) and four representative *Leptospira* species. Translated nucleotide sequences were aligned as described in the text. Amino acids conserved across all sequences are coloured according to the hydrophobic index: dark blue, very hydrophobic; blue, hydrophobic; purple, neutral; red, hydrophilic. The amino acids shown in grey correspond to the acid loop described for the *Leptospira* sp. LipL32 protein by Vivian *et al.* (2009) and Hauk *et al.* (2009).
